# Action Processing and Mirror Neuron Function in Patients with Amyotrophic Lateral Sclerosis: An fMRI Study

**DOI:** 10.1371/journal.pone.0119862

**Published:** 2015-04-17

**Authors:** Laura Jelsone-Swain, Carol Persad, David Burkard, Robert C. Welsh

**Affiliations:** 1 Department of Psychology, University of South Carolina Aiken, Aiken, SC, United States of America; 2 Department of Radiology, University of Michigan, Ann Arbor, MI, United States of America; 3 Department of Psychiatry, University of Michigan, Ann Arbor, MI, United States of America; UCLA, UNITED STATES

## Abstract

Amyotrophic lateral sclerosis (ALS) is a highly debilitating and rapidly fatal neurodegenerative disease. It has been suggested that social cognition may be affected, such as impairment in theory of mind (ToM) ability. Despite these findings, research in this area is scarce and the investigation of neural mechanisms behind such impairment is absent. Nineteen patients with ALS and eighteen healthy controls participated in this study. Because the mirror neuron system (MNS) is thought to be involved in theory of mind, we first implemented a straightforward action-execution and observation task to assess basic MNS function. Second, we examined the social-cognitive ability to understand actions of others, which is a component of ToM. We used fMRI to assess BOLD activity differences between groups during both experiments. Theory of mind was also measured behaviorally using the Reading the Mind in the Eyes test (RME). ALS patients displayed greater BOLD activity during the action-execution and observation task, especially throughout right anterior cortical regions. These areas included the right inferior operculum, premotor and primary motor regions, and left inferior parietal lobe. A conjunction analysis showed significantly more co-activated voxels during both the observation and action-execution conditions in the patient group throughout MNS regions. These results support a compensatory response in the MNS during action processing. In the action understanding experiment, healthy controls performed better behaviorally and subsequently recruited greater regions of activity throughout the prefrontal cortex and middle temporal gyrus. Lastly, action understanding performance was able to cluster patients with ALS into high and lower performing groups, which then differentiated RME performance. Collectively, these data suggest that social cognition, particularly theory of mind, may be affected in a subset of patients with ALS. This impairment may be related to functioning of the MNS and other regions related to action processing and understanding. Implications for future research are discussed.

## Introduction

Amyotrophic lateral sclerosis (ALS) is a progressive neurodegenerative disorder affecting both upper and lower motor neurons of the brain and spinal cord. The disease affects motor functioning, resulting in paralysis and eventual death typically from respiratory failure. There is increasing evidence that patients with ALS exhibit signs of multi-modal dysfunction, even in the early stages. Such impairments include cognitive [1–7] and behavioral (e.g. apathy and social disinhibition) dysfunction [2,8–14]. Previous population-based studies have estimated approximately 35% of patients with ALS have these impairments, and an estimated 15% also have frontotemporal dementia (ALS-FTD) [2,7]. Although psychological symptoms in ALS were reported more than 80 years ago [15], the characterization of cognitive dysfunction in this disease remains poorly understood.

Recent evidence suggests that theory of mind (ToM), which is a higher level social-cognitive ability, may be one of the cognitive domains affected in ALS [16–18]. ToM describes ‘the ability to be in another person’s shoes’, for example by inferring the state or thoughts of another person. Understanding the actions of others is one domain of ToM function. By simply observing another person’s motor movement (e.g. reaching for the cup), one is able to interpret the meaning of that movement (e.g. to pick up the cup) and even propose the intent and goal of the action (e.g. to drink and quench thirst) [19,20]. The action observation network (AON) is recruited during this behavior, which has been associated with the location of mirror neurons (and is sometimes described interchangeably as the mirror neuron system (MNS) [19]). It is suggested that this process of understanding motor actions of others is established by covertly mapping these actions onto one’s own motor repertoire, as if mentally simulating the action [21]. Action understanding is therefore a relevant research area in the field of ALS because motor regions in the brain progressively degenerate, therefore the question remains whether the ability to process or understand the movements of others also becomes affected.

Mirror neurons were first identified in the ventral premotor cortex and inferior parietal lobule [22–25] in the macaque monkey using single-cell recording. The landmark finding from these studies was that the same neurons became excited when the primate executed an overt motor action (e.g., reaching for an object), as well as when they observed the same action performed by another individual. Mirror neurons have since been recognized in humans and are believed to serve a role in social-cognition [20,26–30]. Importantly, these neurons may be involved in the progression of ALS [31,32].

To investigate the ability of patients with ALS to process or understand the actions of others, we examined motor mirror neuron function using functional magnetic resonance imaging (fMRI). At the first level, we aimed to determine if the basic underlying neural substrate of the MNS system is affected. This was investigated during a straightforward motor action-execution and observation experiment. Our second aim was to examine neural and behavioral responses during social-cognitive ToM processing, specifically understanding the actions of others. Theory of mind was further evaluated behaviorally using a standardized assessment.

We hypothesized that patients with ALS would show greater activation compared to healthy controls throughout regions in the MNS during both experiments, particularly the inferior frontal gyrus and inferior parietal lobe. We additionally hypothesized that in the second experiment we would see increased recruitment in prefrontal and posterior parietal regions that are involved during motor simulation states (such as theory of mind [33]) and recognition of pantomimed movement [34,35]. Clinically, the MNS is believed to be important during social behavior (*see* [36,37]; thus any alteration of this capacity (behaviorally, cognitively, or physiologically) in ALS may be important in clinical management and care.

## Methods

### Participants

Nineteen patients with ALS participated in this study. Patients were recruited through the University of Michigan ALS Clinic and were all diagnosed by a neuromuscular physician specializing in ALS with either probable or definite ALS, according to the El Escorial Criteria. Twenty healthy controls (HCs) were matched for age and gender and were recruited through the general Ann Arbor community through the www.umclinicaltrials.org website and from flyer postings.

Participants were without contraindication to MRI and able to lie on their back for over one hour without respiratory distress. Exclusion criteria included a current diagnosis of a psychological or neurological disorder (other than ALS), or self-reported history of drug or alcohol abuse. Additionally, healthy control participants were excluded if they showed signs of depression or mild cognitive impairment, resulting in exclusion of two controls. Two HCs were unable to complete the fMRI portion of the study due to claustrophobia while in the magnet. These two participants completed all other parts of the study.

Patients with ALS completed the ALS Functional Rating Scale, revised version (ALSFRS-R), which is a validated instrument of disease severity [38]. The maximum possible score on this scale is 48, with lower scores indicating greater disease severity. Mean age, gender, mean years-of-education (based on degree obtained), mean months-since-symptom-onset, and ALSFRS-R means scores are presented in Table 1.

**Table 1:** Demographic Information for ALS and Healthy Control Groups.

Demographic Variables	ALS patients (n = 19) Mean (SD)	Healthy controls (n = 18) Mean (SD)
Age	57.21 (8.74)	59.86 (8.63)
Sex	13M, 6F	11M, 7F
Education years	13.58 (2.09)	16.28 (2.35)
MSO	47.00 (33.68)	NA
ALSFRS-R bulbar	10.89 (1.37)	NA
ALSFRS-R limb	14.84 (5.35)	NA
ALSFRS-R resp.	11.05 (1.54)	NA
ALSFRS-R total	36.84 (6.53)	NA

Education Years = years-of-education based on degrees obtained; MSO = months-since-symptom onset; ALSFRS-R bulbar = sub-score from ALS Functional Rating Scale-Revised questionnaire evaluating bulbar function (questions 1–3); ALSFRS-R limb = sub-score from ALS Functional Rating Scale-Revised questionnaire evaluating limb function (questions 4–9); ALSFRS-R resp. = sub score from ALS Functional Rating Scale-Revised questionnaire evaluating respiratory function (questions 10–12); ALSFRS-R total = total score of ALS Functional Rating Scale-Revised questionnaire.

Education was calculated based on highest degree obtained. Specifically, a high school diploma or GED equaled 12 years; an associate’s or trade degree equaled 14 years; a bachelor’s degree equaled 16 years; a master’s degree equaled 18 years; a doctoral degree (medical, philosophy, or equivalent) equaled 20 years. Additional years of education beyond each degree but without obtaining the next level of degree added one year to the education level. This was calculated so education of a person many years of college, but without obtaining a degree, was not equivalent to someone who had the same years of college but with an appropriate degree.

#### Ethics Statement

This study was approved by the Institutional Review Board at the University of Michigan (Study: HUM00053092). All participants provided written informed consent prior to participation. No participants were mentally disabled; therefore, all had the capacity to provide consent. If a patient was unable to produce a written signature, a verbal consent was given and a legal representative consented in writing on the behalf of participant.

### Neuropsychological Assessment

Cognition between groups was assessed using the both the ALS Cognitive Behavior Screen (ALS-CBS) [39] and Montreal Cognitive Assessment (MoCA) [40]. The ALS-CBS tests for frontal lobe-mediated abilities, and is sensitive in screening patients with comorbid frontotemporal dementia (FTD). The MoCA is aimed to screen for mild cognitive impairment in general elderly populations, therefore was well suited to screen for cognitive impairment in the healthy control group.

Theory of mind is often related to executive functioning, which involves higher-level cognitive processes such as problem solving, planning, and working memory. To test for executive function differences between groups, standardized behavioral assessments of executive function were conducted. Verbal fluency is among the most sensitive measures of executive dysfunction in ALS [41], therefore the Controlled Oral Word Association Test (COWAT, for letters C, F and L) and the Category verbal fluency (Animal Naming) tasks were used. To adjust for speech impairment, a Verbal Fluency Index score was calculated using the number of words generated and the length of time it took to repeat these words [42].

A standardized behavioral measure of theory of mind processing was also implemented, specifically the Reading the Mind in the Eyes Test (RME) [43]. The RME assesses a person’s ability to infer the emotions of others by examining pictures of facial eye expressions. In this test, participants are shown a picture in which only the region of the face surrounding the eyes is displayed. Participants are then asked to select a phrase (out of four choices) that best describes the way the person in each picture is feeling.

Behavioral changes in the ALS patients were examined with two different screens: the ALS-CBS caregiver form and the Emotional Lability Questionnaire. The ALS-CBS caregiver form [44] is a questionnaire for the patient’s caregiver to report the amount of behavioral change observed since the onset of the patient’s ALS symptoms, such as apathy and depression. Lower scores indicate greater behavioral change. Pseudobulbar affect, which includes uncontrollable bouts of crying or laughing, was measured using the Emotional Lability Questionnaire (higher scores reflect more symptoms) [45]. Both the patient and the caregiver completed this questionnaire. Lastly, depressive symptoms were measured using the Geriatric Depression Scale, shortened version [46], which screening tool for depression. This measure was chosen because it does not incorporate questions that could confound depressive symptoms with physical or vegetative symptoms specific to ALS, such as changes in sleeping or eating patterns. Higher scores on this screen suggest greater risk of depression. All neuropsychological measurements are described in Table 2.

**Table 2:** Descriptive results of neuropsychological assessments administered to patients with ALS and healthy controls.

Tests/Screen Domains	Behavioral Measure	Neuropsychological Test Measurement	ALS Patients (n = 19) Mean (SD)	Healthy Controls (n = 18) Mean (SD)
Executive Function Tests	Verbal Fluency	COWAT VFI	4.88 (1.37)	4.46 (0.95)
		*Animal Naming VFI	2.83 (0.71)	2.40 (0.47)
	Theory of Mind	*Reading the Mind in the Eyes Test (% accurate)	74.88 (9.10)	70.60 (6.40)
	
Cognitive/behavioral Impairment Screens	Global Cognition	MoCA	24.63 (3.09)	25.83 (1.95)
	Frontal Lobe Function	ALS-CBS	15.52 (2.44)	16.00 (1.90)
		ALS-CBS Caregiver Form (ALS n = 16)	33.56 (7.85)	NA
Pseudobulbar Affect Screen	Emotional Lability	ELQ	4.47 (8.75)	NA
		ELQ Family (ALS n = 15)	6.47 (10.75)	NA
Mood	Depressive Symptoms	Geriatric Depression Scale, short version	4.26 (3.71)	0.78 (1.40)


COWAT = Controlled Oral Word Association Test; VFI = verbal fluency index; MoCA = Montreal Cognitive Assessment; ALS-CBS = ALS Cognitive Behavioral Screen; ELQ = Emotional Lability Questionnaire from person with ALS; ELQ Family = Emotional Lability Questionnaire from relatives and caregivers.

### fMRI Protocol

Blood oxygenated level dependency (BOLD) signal activity was collected over a period of 8.9 minutes for Experiment 1 and 12.0 minutes for Experiment 2. Both experiments were implemented using E-Prime v. 2.0. These experiments were projected onto a screen at the head of the scanner bore and viewed with a back-projected mirror placed on the head coil.

#### Experiment 1: Action Observation and Execution

A key property of mirror neurons is involvement during observation of an action [47]. Therefore, to test whether mirror neurons may be affected in ALS, the first experiment implemented a basic task involving the simple observation of actions. This experiment involved a block design that included two alternating conditions: i) an action-observation condition (watching a video on a computer screen of someone squeezing a ball), and ii) an action-execution condition (the participant squeezing a ball). The action-execution component served as a benchmark for replicating previous research. Previous research has shown that BOLD signal differences in patients with ALS during motor execution resulted in greater activity in the motor regions, even after correcting for effort [48]. Therefore, we expected to see similar activity differences in the observation condition, supporting our hypothesis that mirror neuron function is affected in ALS. Fourteen blocks total were presented, alternating between observe and execute conditions. These blocks were separated by a jittered fixation (between 4-8sec) and were preceded by a 1sec prompt of either the word “watch” or “squeeze”.

**Fig 1:**
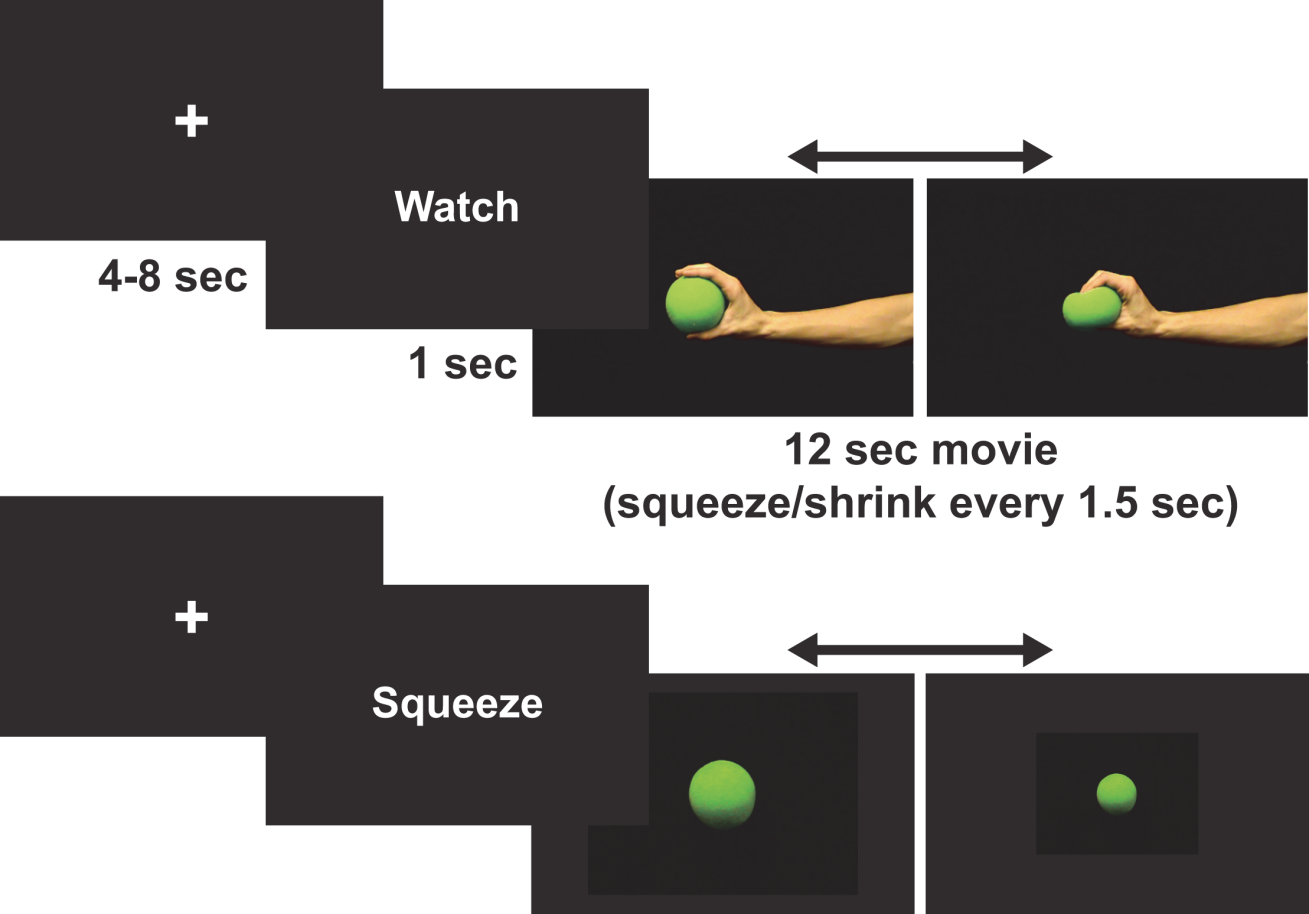
Example of a trial from each condition (action-observation and action-execution) in Experiment 1.

During the observe condition, participants watched an actor’s hand rhythmically squeeze a ball every 1.5 seconds over a 12sec-block duration (see Fig 1). Participants were told to passively observe this action and to not move their own hand while observing. Participants were monitored from outside the scanner to ensure these instructions were followed.

During the action-execution condition, participants squeezed a small soft ball every 1.5sec for a duration of 12sec using their dominant/non-affected hand. During this time participants viewed a picture of a ball on the computer screen that shrank every 1.5sec to signal when to squeeze their hand. Participants were not shown an actor’s hand in this condition to reduce the confound of activity that would be induced by action observation [49]. Participants were told to squeeze the ball at a comfortable level in order to reduce expected increased exertion and neural recruitment by patients with ALS [48,50–52].

The number of squeezes observed and executed were the same within scans and between participants. To control for tactile stimulation, participants held the ball throughout the duration of the scan. To assist with hand weakness for ALS participants, Velcro straps were wrapped around the ball and the hand of the participant to hold the ball in place. For experimental consistency, this was done for every participant. Participants were trained outside the scanner on this experiment.

#### Experiment 2: Action Understanding

Experiment 2 was carried out to examine involvement of the mirror neuron network during motor cognition, specifically action understanding. This experiment was a modified version of that designed by Molenberghs et al. [27]. In Molenberghs et al. [27], three conditions were examined in order to tease apart context effects. The goal of the current study, however, was to measure activity during action understanding, which was compared to passive observation of actions. Therefore our experiment only included two of the three conditions from Molenberghs et al. [27]. Another difference was that only one run was implemented (opposed to two) to reduce possible fatigue or discomfort for the patients.

**Fig 2:**
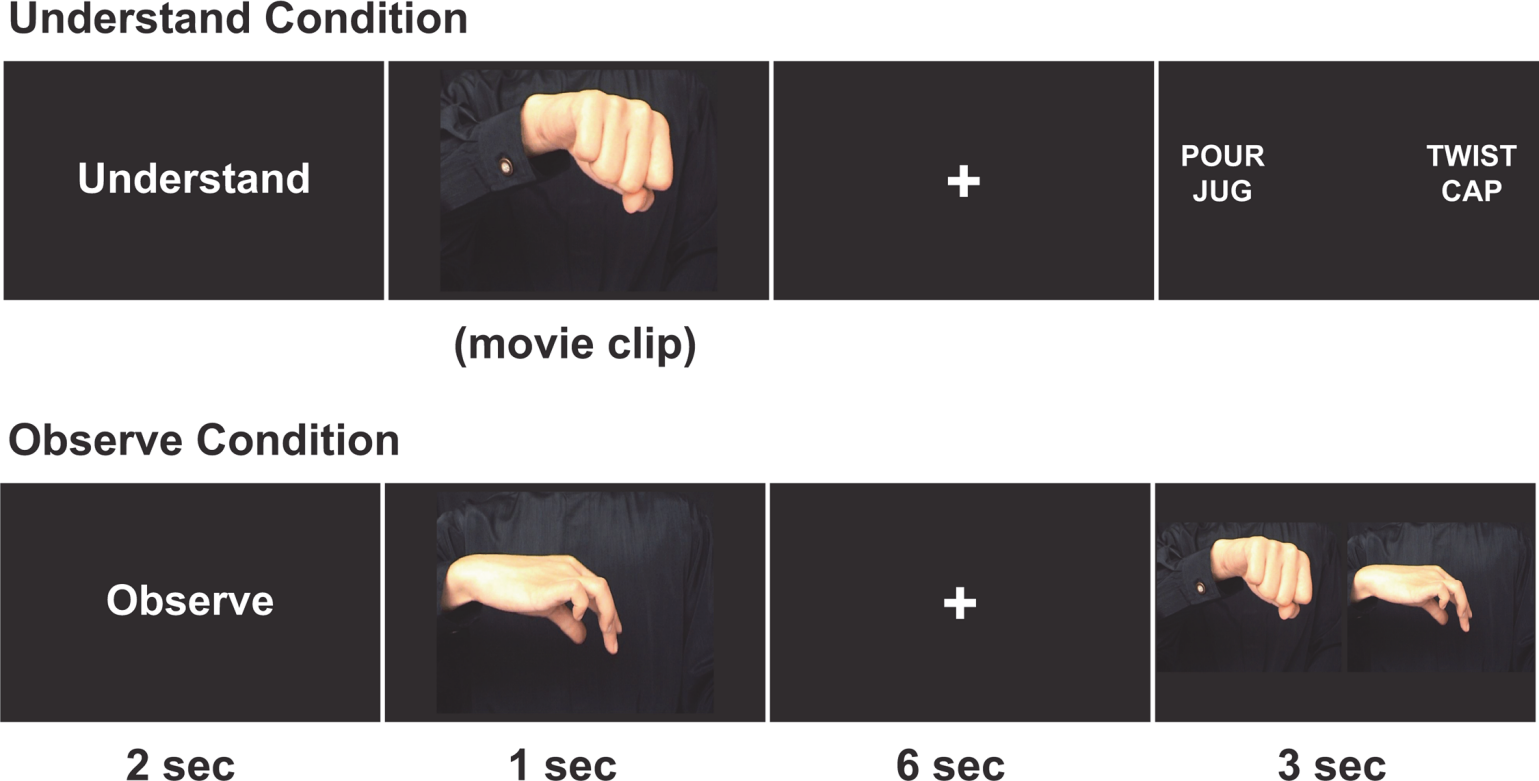
Example of a trial from each condition (understand and observe) in Experiment 2.

During this experiment, participants watched a computer screen and were told to either passively observe (observe condition) or actively understand (understand condition) a short (1sec) video of an actor pantomiming an action with their hands. In each video, only the torso section of the actor was displayed and no objects were used (*see* [27]). Each video was preceded by the condition cue (“observe” or “understand”) and followed by a 6sec delay. After the delay, participants were either shown a 2sec no-go (catch trial) screen or a response screen. During the observe condition, the response screen showed two side-by-side pictures. Participants had to select which picture matched a still-frame scene from the video. During the understand condition, participants had to select the correct action from two phrases (see Fig 2). There were 20 trials of each condition, randomly presented per participant.

During this scan, all participants held in each hand a small response bulb that was Velcro-strapped around their palms (to assist with hand weakness). Participants made a forced-choice response by either hand, corresponding to the location of their response selection on the screen (left or right). Because some patients were not able to make a physical response, all participants were also instructed to think of their selection.

### Imaging Acquisition

All scanning was performed at the University of Michigan's Functional MRI Laboratory on a GE 3T Excite 2 (General Electric, Milwaukee, WI, USA). During each participant's session, medium-resolution spin-echo (T_1_-Overlay) and high-resolution spoiled-gradient recall (T_1_-SPGR) anatomic images were collected in the axial plane (256 x 256 matrix, 220 mm FOV, and with 1.2 mm slice thickness). T_1_-Overlay images were acquired in the same-slice locations as the T2* volumes, however at a higher in-plane resolution (256 x 256 matrix, 220 mm FOV). T2* time-series data were acquired in the axial plane (aligned to the anterior–posterior commissure) using a reverse-spiral k-space readout. A total of 266 and 360 T2*-weighted volumes were collected for each participant during Experiment 1 and Experiment 2, respectively (repetition time TR = 2sec, 40-slice volumes at 3 mm slice thickness and no skip, echo-time TE = 30ms, 64 x 64 matrix, 220 mm FOV). Four T2* volumes at the beginning of each time-series acquisition were excited but not recorded in order to achieve thermal equilibrium of magnetization.

### Preprocessing

Slice timing and motion correction were applied using Statistical Parametric Mapping, version 8 (SPM8; Wellcome Trust Center for Neuroimaging, (http://www.fil.ion.ucl.ac.uk/spm, release 4667) and FSL's MCFLIRT (http://www.fmrib.ox.ac.uk/fsl version 5.0.2.2), respectively. Each participant's T_1_-Overlay volume was co-registered to the time-series data; the T_1_-SPGR was then co-registered to the co-registered T_1_-overlay image. Spatial normalization to the Montreal Neurological Institute (MNI 152) template of the resulting co-registered T_1_-SPGR image was then performed using SPM8/VBM8. The resulting normalization was applied to the slice-time-corrected time-series data. The resulting T2* images had 3 mm isotropic voxels. These normalized T2* time-series data were subsequently spatially smoothed with a 5 mm Gaussian kernel.

### fMRI Data Analyses

All fMRI statistical models were conducted using SPM8. In Experiment 1 (action observation and execution), fMRI blood oxygenated level dependency (BOLD) signal differences were analyzed using a standard hemodynamic response function, implemented with the General Linear Model (GLM). In this task, first level regressor estimates for each of the conditions (observe and execute) were obtained by contrasting each condition (observe, execute, observe + execute) against the implicit resting baseline (contrasts [1, 0, 0]; [0, 1, 0]; [.5,. 5, 0]). Second-level random-effects analyses were compared between ALS patients and HCs using uncorrected *p* < 0.001 threshold.

The primary goal of this experiment was to examine the basic functioning of the MNS in patients with ALS. Again, the MNS is composed of neurons that respond to both an executed action and observation of that same action. Therefore a conjunction analysis was performed to extract the intersected (overlapping) voxels within the MNS that were activated specifically during both action observation and execution conditions. An inclusion mask of MNS regions identified in the human brain (*see* [53]) was created using the WFU PickAtlas software tool [54] and was applied at the second-level for Experiment 1. Overlapping voxels of activity resulting from these two conditions within the MNS mask were counted per participant using a custom code written in MATLAB, and then compared between groups using an independent sample *t*-test.

In Experiment 2 (action understanding), first level analyses were run using the finite response function (FIR), following in line with methods implemented by Molenberghs and colleagues [27]. In this model, only neural activity resulting from observing or understanding the action in each trial was included in the analysis, therefore not confounding results with activity derived from the overt motor response made after each trial. To examine activity specific to the social cognitive task of understanding actions, first-level analyses were run with the *understand > observe* contrast (1–1 0) using the GLM. This was entered into a second-level random-effects group whole brain analysis between ALS patients and HCs using an uncorrected *p* < 0.001 threshold.

### Experiment 2 Behavioral Data: Action Understanding

Accuracy data was collected in Experiment 2 for understand and observe trials and was compared between patients with ALS and HCs. Omissions were not included in accuracy measurements. Comparisons between groups were made using independent sample *t*-tests in SPSS, version 22.

## Results

### Neuropsychological Assessments

Means and standard deviations for each of the cognitive and social measurements that were gathered (ALS Cognitive Behavioral Screen, Montreal Cognitive Assessment, Controlled Oral Word Association Test, Categorical Fluency for Animals, Reading the Mind in the Eyes, Emotional Lability Screen, and the Geriatric Depression Scale, shortened version) are reported in Table 2 for both patients with ALS and healthy controls. One patient scored low on the MoCA, but no participant scored below the cut-off for frontotemporal dementia on the ALS-CBS (therefore indicating a low risk of participants with FTD). Two patients showed signs of pseudobulbar affect according to the ELQ (from both self and caregiver reports), and both patients’ caregivers reported high levels of behavioral changes according to the ALS-CBS caregiver form. Neither of these patients appeared at high risk for cognitive dysfunction (e.g. one patient scored in the 96^th^ percentile on the COWAT). One patient was at risk for having depression, according to the GDI. One healthy control scored below the 5^th^ percentile during the verbal fluency tests and scored in the cognitively impaired range on the MoCA, and another scored in the range for suspected depression on the GDI. Accordingly, data from these control participants were removed from the study and not included in any of the analyses or tables.

Between-group differences for verbal fluency (COWAT), general cognition (ALS-CBS and MoCA), and theory of mind (RME) were tested. Because scores between the COWAT and Animals were collinear (Pearson’s *r* = 0.36, *p* = 0.03), only the COWAT was included in the model since this test is more represented in the literature. To account for multiple comparisons between tests and to control for level of education (*t*(35) = 3.70, *p* < 0.01, see Table 1), a multivariate analysis of covariance (MANCOVA) was conducted. One patient and one HC did not complete the RME, resulting in 18 patients and 17 HCs in this analysis. Overall, there was not a main effect of group, *F(*1, 31) = 0.60, *p* = 0.71, however the effect of education reached near significance, *F*(1, 31) = 2.60, *p* = 0.05. This model showed no univariate differences in any of the neuropsychological assessments between patients with ALS and HCs, however, education had a significant effect on the verbal fluency test (COWAT, *p* = 0.02). These results imply the groups were overall similar in their performance but that level of education (regardless of group) was related to verbal fluency ability.

### Experiment 1: Action Observation and Execution

#### fMRI Data

**Fig 3:**
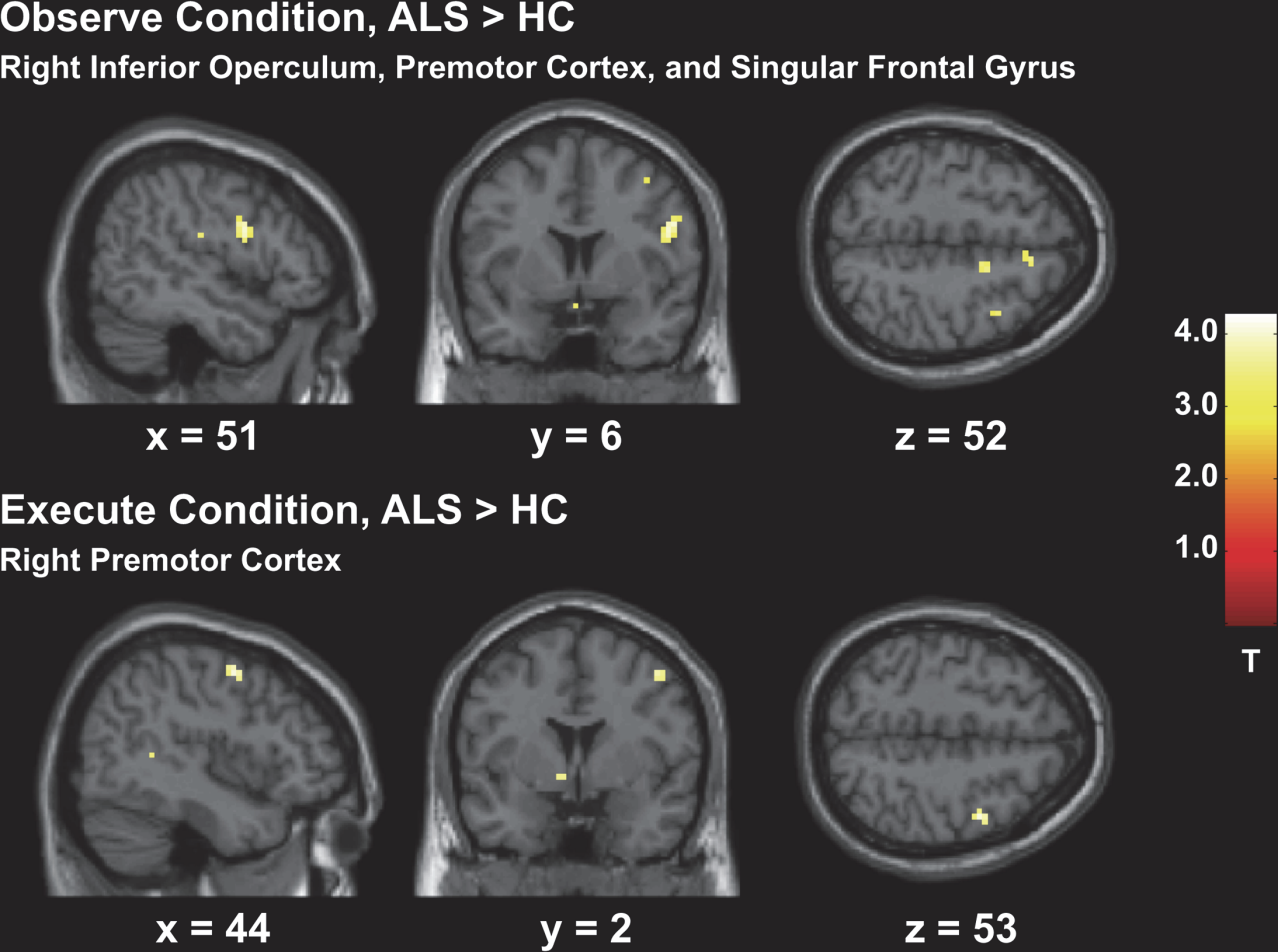
Experiment 1 Action Observation and Execution Task, ALS > HC. Greater activity in the ALS group compared to HCs throughout the frontal lobe during the action observation and execution conditions.)

**Fig 4:**
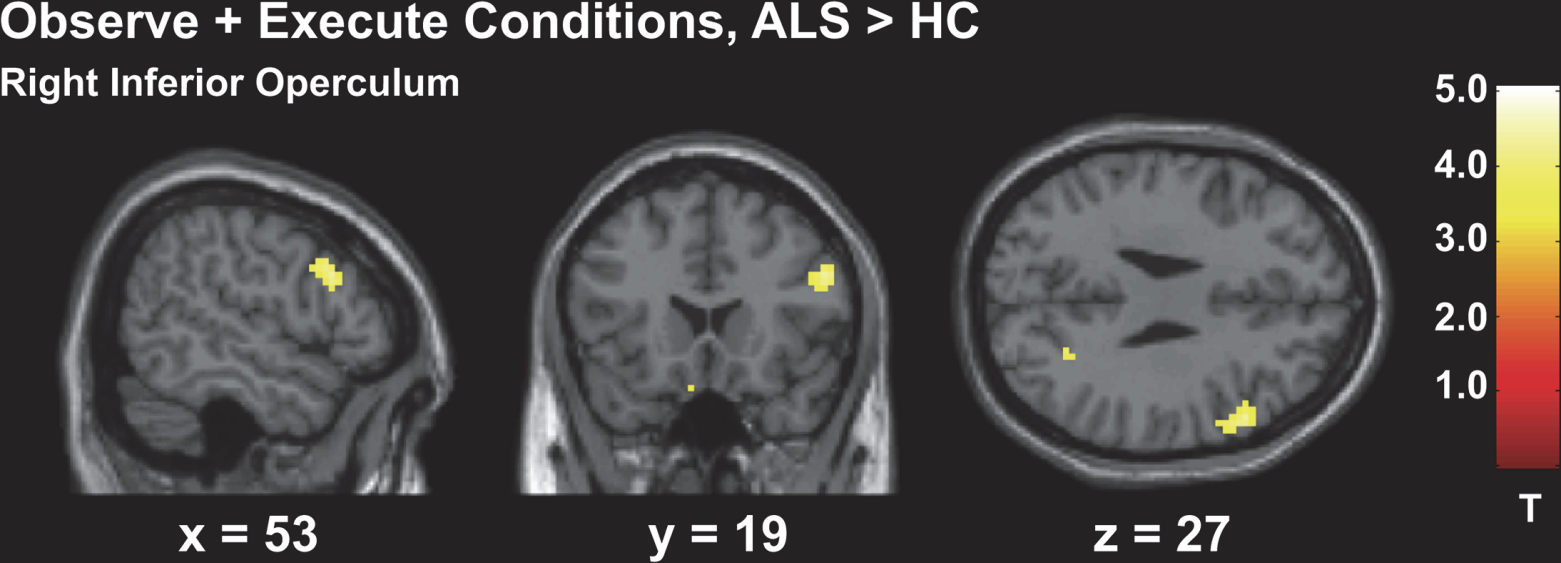
Experiment 1 Action Observation and Execution Task, ALS > HC. Greater activity in the ALS group compared to HCs in the right inferior operculum from the combination of both action observation and execution conditions.)

Nineteen patients with ALS and sixteen HCs were included in these results. Between-group analyses in Experiment 1 showed greater activity in the ALS group compared to HCs for all three contrast conditions. Greater activity was observed throughout the MNS, particularly in the right hemisphere. Notably, the right inferior operculum was more active in the ALS group for all three contrasts (*observe*, *execute*, *observe + execute*), *p* < 0.001 (uncorrected, see Figs 3 and 4). There was also greater activity in left inferior parietal lobe and left middle temporal gyrus during both the *observe* and *observe + execute* contrasts. The main condition of interest, the observe condition, resulted in increased activity throughout the right frontal lobe including the supplementary motor area and rolandic operculum, the right parietal and temporal lobes, and the basal ganglia and cerebellum. The *observe + execute* contrast also recruited greater right frontal, parietal, and superior temporal gyrus activity as well as increased activity in the left primary motor cortex, left subcortical regions, and cerebellum. In accordance with previous literature, the motor execution condition resulted in greater activity in the ALS group compared to HCs. Specifically, activity was greater in the right premotor region (see Fig 3), right somatosensory cortex, right middle temporal gyrus, and left basal ganglia. The HC group exhibited no regions of greater activity compared to the ALS group in any contrast. See Table 3 for complete results.

**Table 3:** Anatomical regions and coordinates located in activation maps during Experiment 1 (Action Observation and Execution).

					MNI Coordinates		
Experimental Contrast	Group Contrast	Anatomical Region	Cluster Region	t-value	X	Y	Z
**Execute**	**ALS>HC**	Frontal Lobe	R Inferior Operculum	3.67	57	14	31
			R Pre-motor	3.97	45	-1	52
		Parietal Lobe	R Postcentral Gyrus	4.27	63	-16	46
		Temporal Lobe	R Middle Gyrus	3.39	45	-46	7
		Occipital Lobe	L Inferior	3.77	-48	-70	-8
		Basal Ganglia	L Pallidum	3.57	-9	2	-5
			L Caudate	3.48	-9	17	19
**Observe**	**ALS>HC**	Frontal Lobe	R Inferior Operculum	4.26	51	5	25
				3.55	57	14	31
			R Rolandic Operculum	3.97	48	-19	22
			R Supplementary Motor Cortex	3.79	15	2	52
				3.82	6	23	55
			R Middle Gyrus	3.61	39	8	52
			R Superior Gyrus, Orbital	3.41	15	23	-23
			R Superior Gyrus	3.67	15	59	25
			L Superior Gyrus, medial	3.63	-18	65	28
					-3	47	40
			L Inferior Triangularis	3.41	-30	26	19
		Parietal Lobe	R Angular Gyrus	3.48	63	-58	28
			R Precuneus	4.63	24	-61	28
			R Postcentral Gyrus	3.61	63	-19	46
			L Inferior	3.46	-57	-52	49
			L Precuneus	3.53	-12	-58	34
		Temporal Lobe	R Superior Gryus	3.74	42	-40	10
			R Pole	3.70	33	26	-29
			R Fusiform Gyrus	3.75	42	-13	-26
				3.47	42	-31	-29
			L Middle Gyrus	3.72	-57	-28	-17
		Cingulate	R Anterior	3.62	6	29	-2
				3.75	18	29	22
			L Middle	3.50	-15	23	34
		Subcortex	R Basal Ganglia (Caudate)	3.57	15	2	28
			L Basal Ganglia (Pallidum)	3.69	-21	-7	-8
			L Thalamus	3.45	-9	-16	1
				3.45	-6	-10	4
		Cerebellum	Vermis	3.67	0	-55	-2
					-3	-52	-29
			L Crus	3.71	-15	-85	-26
**Execute + Observe**	**ALS>HC**	Frontal Lobe	R Inferior Operculum	4.29	54	20	28
			R Inferior Orbital	3.69	33	35	-2
				3.65	51	29	-20
			R Superior Orbital	3.68	18	17	-23
			R Middle Gyrus	3.53	42	-1	52
			L Middle Gyrus	5.05	-36	53	25
			L Precentral Gyrus	3.70	-51	11	46
		Parietal Lobe	R Precuneus	3.73	24	-61	28
			R Postcentral Gyrus	3.49	57	-25	55
				4.67	63	-19	46
			L Precuneus	3.73	-9	-73	58
			L Inferior	3.86	-57	-55	49
		Temporal Lobe	R Superior Gyrus	3.81	42	-43	7
			L Middle Gyrus	3.88	-60	-28	-17
		Subcortex	L Basal Ganglia (Caudate)	3.87	-9	17	19
			L Thalamus	3.66	-6	-16	-11
			L Parrahippocampal Area	3.63	-15	8	-26
		Cerebellum	R Crus 1	3.55	36	-64	-41
			R Crus 2	3.43	39	-70	-38

R = right; L = left.

Small volume correction analyses were evaluated for specific *a priori* regions of interest in the observe condition using a 5mm sphere, including the inferior operculum and inferior parietal lobe. The right inferior operculum, *p* < 0.001 (MNI: 52, 5, 25), and left inferior parietal lobe, *p* < 0.009 (MNI: -57, -52, 49) were both significantly more active in the ALS group during this task.

**Fig 5:**
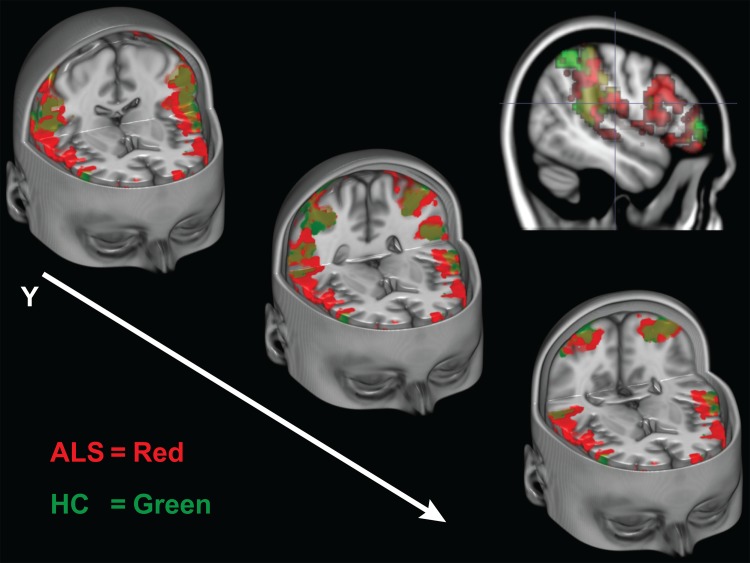
Experiment 1 Conjunction Analysis of Co-activated Voxels During Execution and Observation of Actions. Cut-out sections of the inferior frontal gyri and inferior parietal lobes. Activity maps indicate only the voxels that were active during both the action execution and observation conditions during Experiment 1. The ALS group’s intersecting voxels are shown in red, the HC group is shown is green. Colors are blended in overlapping regions.)

In the conjunction analysis, the ALS group (*M*_*voxels*_ = 505.12, *SE* = 127.70) had significantly greater number of coactivating voxels within the MNS compared to the HC group (*M*_*voxels*_ = 192.06, *SE* = 66.57), *t*(33) = 2.10, *p* = 0.048. This greater extent of activity (see Fig 5) seen in patients with ALS suggests a compensatory mechanism in the ALS brain when both observing and executing an action.

### Experiment 2: Action Understanding

#### fMRI Data

**Fig 6:**
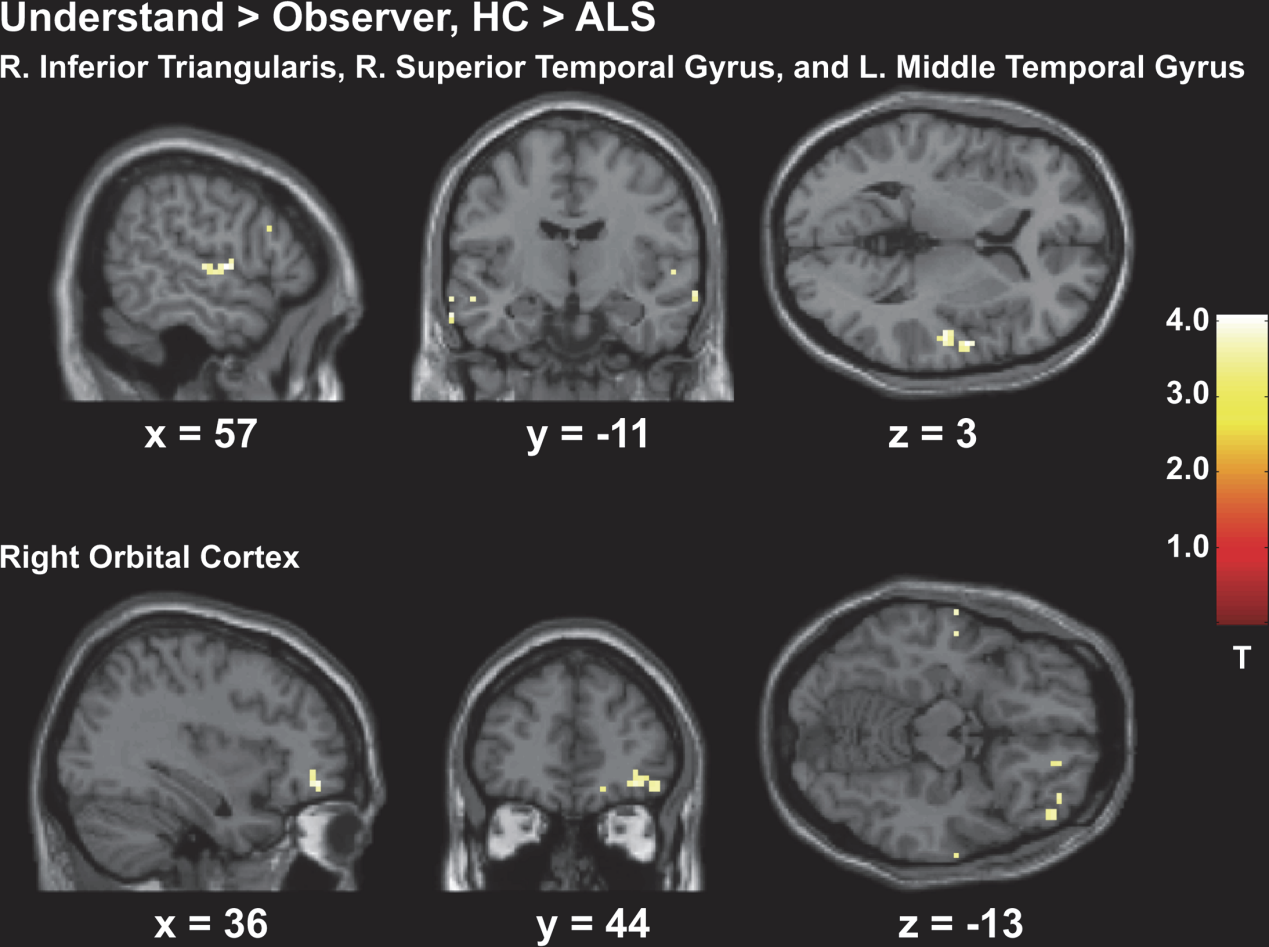
Experiment 2 Action Understand > Observe, HC > ALS. Greater activity in the HC group compared to patients with ALS throughout the right prefrontal cortex and temporal regions shown for the *understand>observe* contrast from Experiment 2.)

Data presented for this experiment includes 18 patients with ALS (one patient was excluded from the analyses because they fell asleep during this experiment) and 16 HCs. Activity from the *understand > observe* contrast displayed greater activity in the HC group throughout right prefrontal cortex including the triangularis, bilateral orbital regions, bilateral temporal lobe, and the occipital lobe (*p* < 0.001, uncorrected, see Fig 6). Regions of greater activity in the ALS group only included the right occipital lobe (See Table 4).

**Table 4:** Anatomical regions and coordinates located in activation maps during Experiment 2 (Action Understanding) for the *understand > observe* contrast.

				MNI Coordinates		
Group Contrast	Anatomical Region	Cluster Region	t-value	X	Y	Z
HC > ALS	Frontal Lobe	R Inferior Triangularis	4.02	60	23	25
		R Inferior Orbital	3.59	48	44	-14
		R Middle Orbital	3.86	36	47	-11
		R Superior Orbital	3.41	18	47	-14
		L Inferior Orbital	3.25	-51	32	-17
	Parietal Lobe	L Postcentral Gyrus	3.43	-27	-34	64
	Temporal Lobe	R Superior Gyrus	4.07	57	-4	4
			3.66	69	-10	-11
		L Middle Gyrus	3.87	-66	-10	-23
			3.66	-66	-10	-14
			3.83	-57	8	-23
			3.59	-54	-10	-14
	OccipitalLobe	L Middle Gyrus	3.84	-36	-61	10
		Calcarine Sulcus	3.37	24	-64	10
ALS > HC	Occipital Lobe	R Inferior Gyrus	3.38	33	-85	-5

R = right; L = left.

To make sure differences in education were not driving these results, we analyzed this data again including years-of-education in the GLM model as a covariate for the *HC > ALS* contrast. Results confirmed that the pattern of BOLD activity differences described above were not a result of education differences between groups. Specifically, the pattern of greater activity in the HC group was similar to that described above, including increased activity in the right frontal inferior operculum and triangularis, right inferior, middle and superior orbital regions, superior and middle frontal gyri, bilateral middle temporal gyri, right superior temporal gyrus, left inferior temporal gyrus, left middle cingulate, left thalamus, and right putamen (*p* = 0.001, uncorrected).

#### Behavioral Data

Behavioral data was obtained for thirteen patients from the ALS group (missing due to motor impairment), and data from three HCs were not collected because of temporary equipment malfunction (response signals were not received by EPrime). A non-parametric Mann-U Whitney test was conducted to compare group accuracy percentage differences. Both groups were similarly accurate in identifying the correct matching picture during the observe condition, *U(24) =* 79, *Z = -*0.28, *p =* 0.80, (*M*_*ALS*_ = 82.65, *SD*_*ALS*_ = 11.14; *M*_*HC*_ = 80.32, *SD*_*H*C_ = 13.79). There was a ceiling effect found for the understand condition in the HC group, and also for a portion of ALS patients, thereby creating a bi-modal distribution of the ALS data.

We therefore separated the ALS patients into two groups according to their action understanding performance (9 patients at ceiling and 6 patients *M* = 78.98(%), *SD* = 10.39). Sub-groups were contrasted at the second level in SPM for the *understand > observe* contrast. It was found that patients who were 100% accurate in their responses also showed greater activity in bilateral frontal superior gyri, whereas patients who performed worse did not have any areas of greater activity (*p* = 0.001, uncorrected).

Because the action understanding task is deemed to assess ToM behavior, we further compared group differences to the standardized Reading the Mind in the Eyes ToM test (RME). Accuracy measurements from the RME test were compared between subgroups using an independent sample Mann-U Whitney Test. It was found that the subgroup of patients who performed better on the action understanding task also performed better on the RME test (*M*_RME_ = 79% accurate) compared to the patients who performed worse (*M*_RME_ = 69% accurate), *U(*12) = 7.5, *Z* = -2.02 *p* < 0.05.

## Discussion

Patients with ALS experience rapid progression of muscle weakness and paralysis. It is known that brain regions responsible for motor execution become compromised. Our study shows more complex motor systems, including the MNS and other extra-motor regions, may also be affected by the disease process.

The first experiment in our study aimed to evaluate basic mirror neuron function by examining neural recruitment within the MNS system when participants either observed or executed a simple motor action. Results from the action execution component supported previous findings of greater recruitment of BOLD activity in patients with ALS [48,50–52]. Importantly, greater activity was recruited in the patient group in MNS regions within the right frontal and left parietal lobes while simply observing a motor action. Both tasks in particular recruited greater activity in the inferior operculum. The operculum is known to be important during action processing [55,56] and is one of the primary regions comprising the MNS [57]. It is suggested the operculum reflects the human homologue to the region F5 in the macaque monkey, where mirror neurons were first identified [22,23,25].

Increased recruitment of the MNS was further supported by the conjunction analysis. When the number of co-activated voxels during both observation and execution of a hand squeeze were examined, patients with ALS had a significantly higher number of intersecting voxels. The conjunction analysis only included voxels that were active during both observation and execution of the same motor action. Therefore, increased voxels seen in the ALS group were partially derived from passive observation of simple actions and not solely a result of motor execution differences. If exertion differences were causing greater recruitment of co-activated voxels, as is typical with motor execution paradigms, then this would infer patients were also exerting greater energy when simply viewing the actions of others. Overall, results from Experiment 1 support the possibility that the MNS is affected by ALS at the basic level, and that the MNS may initiate a compensatory strategy when involved in action processing.

The second experiment in this study examined the higher-level social cognitive ability of action understanding, which is considered a function of the MNS and also a component of theory of mind. This experiment showed that healthy controls were overall better at this task. In line with this behavior they recruited greater activity within the MNS and in regions associated with action understanding, particularly the right frontal regions. Healthy controls also recruited more activity in the left middle temporal gyrus, which has recently been identified as the primary region that bridges together the two neural pathways involved specifically in action understanding [19]. Patients with ALS recruited more activity only in the occipital lobe.

Results from the second experiment do not support a role for compensatory MNS recruitment in ALS. However, results from both studies do suggest there are differentiated roles of the MNS during basic action processing compared to action understanding. Likewise, these results appear to reflect behavioral performance. Specifically, behavioral performance and neural function during simple observation of actions may be more similar to the mechanisms involved during the execution of an action in ALS. This corresponds to mirror neuron literature showing neural responses to both executed and observed actions [58], and also corresponds to literature showing a compensatory strategy initiated by the execution of an action in patients with ALS [48,50,59]. Research shows involvement of neural regions both within and outside the MNS during action understanding [19]. Therefore, better performance by the HC group could be reflected in the greater neural recruitment seen in the action understanding task, which would not counter the possibility of a compensatory MNS theory. Rather, it would support the notion that action understanding may be compromised in ALS, without the resources for neural compensation. This pattern of greater neural recruitment corresponding to better action understanding performance was also seen in the patients who scored at ceiling on this task compared to the lower performing patients.

Results from these experiments, especially the first experiment, share similarities with work examining motor imagery in ALS. For example, increased activity in patients with ALS have been seen during imagined movement throughout the parietal, premotor, and primary motor cortices [59,60], and behavioral differences during imagined hand positions have been reported [61]. Both the action observation network (AON) and motor imagery (MI) network are presumed to fall under a hierarchical simulation network, which share a core network [35]. Specifically, the AON includes bilateral involvement of the premotor cortex, supplementary motor cortex, inferior frontal gyrus, prefrontal cortex, posterior middle temporal gyrus, superior and inferior parietal lobe, and the posterior cingulate gyrus [62]. The MI network however is thought to entail the premotor cortex, supplementary cortex, cingulate, superior, inferior and middle frontal gyri, inferior parietal lobe, basal ganglia, and cerebellum [63].

Motor simulation processes support various motor functions, including recognition and understanding [19], and are important precursors for executing actions. For example, when learning a motor movement, increased activity is seen throughout the extra motor regions within the AON and MI network, and is negatively correlated to skill level (see [64]). This trend of neural efficiency has been demonstrated when learning to sequence strokes on a guitar [49] and also in expert gymnasts [65]. These studies indicate that as one masters a skill, the demand on extra-motor neural resources for action execution decreases. Corroborating research involving imagined and executed movements in ALS [48,50,52,66,67] indicates the opposite effect is happening, specifically as the ability to produce overt motor actions decreases, the need for neural resources increases. However, other research has shown a decrease in activity in motor regions during executed movement [68], which may be specific to a later disease stage process [52]. This emphasizes the need for continued longitudinal evaluation of motor network changes.

As previously stated, motor imagery and action observation networks are considered to be two distinct networks [19,63], yet with considerable overlap [63,69]. However, it is unknown the level of interaction, and whether observation of actions can automatically activate motor imagery [63]. This is a potential confound in the current study, as these systems are possibly intertwined [63]. Interestingly, research comparing these systems is minimal. Specifically, a recent meta-analysis showed that of all the motor imagery and action observation studies, only approximately 3% examined both functions [63].

Although data from this study may suggest a role of mirror neuron involvement and decreased ability to process the actions of others in patients with ALS, other explanations must be considered. It has already been suggested that motor imagery may confound results with motor observation. Results from this study may also support the possibility that a preparatory role in action initiation is compromised [48]. This possibility could explain the increased cortical activation seen in the ALS group during Experiment 1 as opposed to direct mirror neuron involvement. Last, action understanding processing in ALS may be distinct from the mirror neuron system and therefore not directly related to mirror neuron processing [19]. This would suggest a more general role of action processing that encompasses various motor functions being compromised in ALS.

### Reading the Mind in the Eyes Test

Behavioral performance during the theory of mind task (Reading the Mind in the Eyes, RME [43]) was not different between patients with ALS and healthy controls. This supports a previous study that also used the RME [17]. A second research study that used this assessment however found group differences that were trending toward significantly poorer performance by the ALS group [14]. When all three studies are examined collectively, it appears ToM as examined by the RME is not particularly sensitive in ALS. However, other work has found ToM changes in patients with ALS when using other forms of ToM assessment [14,17,18,70]. Particularly, ToM differences may be more pronounced during social situations [17].

In all four previous ToM experiments [14,17,18,70], patterns of behavioral differences became more apparent when patients were examined more closely at the individual level. When examined more closely, our data too showed that patients could be separated according to performance in the action-understanding task. Specifically, patients who performed at ceiling on the action-understanding task also recruited greater activity in the bilateral superior frontal gyrus, supporting a trend of increased recruitment during better action understanding performance that was also seen in the HC group. Additionally, these specific patients performed better in the Reading the Mind in the Eyes (RME) task [43]. Theory of mind performance may therefore be able to further identify patients who display social-cognitive symptoms, yet without global cognitive impairment.

This idea corroborates recent research suggesting ToM could be sensitive to identifying cognitive impairment [14]. It may be possible to even dissociate ToM impairment from other executive function abilities [14], which our data partly supports as patients were not impaired during the executive function task (verbal fluency) in this study. Although executive functions are the most commonly reported cognitive symptoms in ALS [8,39,42,71–73], it is becoming clear that cognitive impairment is not limited to executive functioning. For example, additional domains such as emotional [14,74–76] and sensory processing [77] impairments have been reported in patients with ALS. Even more related to our current study is research showing an impairment in processing action words (verbs) [78–81].

### Limitations

It is noted that several clinical measures that may confound cognitive function were not collected in this study, such as forced vital capacity and behavioral impairment. Both patients who were at risk for behavioral impairment, according to the ALS-CBS, also had scores from the ELQ indicating possible pseudobulbar affect. Neither of these patients however showed any signs of cognitive impairment, and both performed at ceiling on the action understanding task. Another limitation is that neuroimaging results presented are at the uncorrected (*p* < 0.001) threshold, thus warranting further replication. Despite a liberal analysis, results were consistently in accord with brain regions associated with action observation and understanding.

### Conclusions

Overall, this study supports ALS as a multi-system disorder, and furthermore corroborates research showing that social cognitive functioning, and specifically theory of mind, can be negatively impacted in some patients. It would be of interest to examine whether action observation would help prolong extra motor network function [82] and slow the progression toward previously observed decreased neural recruitment [52]. Further research is needed to explore the specific involvement mirror neurons may play in ALS, and how this may aid in furthering our understanding of the pathogenesis of this disease.

## References

[1] Abrahams S, Goldstein LH, Simmons A, Brammer M, Williams SCR, Giampietro V, et al Word retrieval in amyotrophic lateral sclerosis: a functional magnetic resonance imaging study. Brain 2004;127:1507–17. 15163610 10.1093/brain/awh170

[2] Montuschi A, Iazzolino B, Calvo A, Moglia C, Lopiano L, Restagno G, et al. Cognitive correlates in amyotrophic lateral sclerosis: a population-based study in Italy. Journal of Neurology, Neurosurgery & Psychiatry 2014; 10.1136/jnnp-2013-307223 24769471

[3] Hanagasi HA, Gurvit IH, Ermutlu N, Kaptanoglu G, Karamursel S, Idrisoglu HA, et al Cognitive impairment in amyotrophic lateral sclerosis: evidence from neuropsychological investigation and event-related potentials. Brain Res Cogn Brain Res 2002;14:234–44. 12067696 10.1016/s0926-6410(02)00110-6

[4] Merrilees J, Klapper J, Murphy J, Lomen-Hoerth C, Miller BL. Cognitive and behavioral challenges in caring for patients with frontotemporal dementia and amyotrophic lateral sclerosis. Amyotroph Lateral Scler 2010;11:298–302. 10.3109/17482961003605788 20222805 PMC2908374

[5] Giordana MT, Ferrero P, Grifoni S, Pellerino A, Naldi A, Montuschi A. Dementia and cognitive impairment in amyotrophic lateral sclerosis: a review. Neurol Sci 2010;32:9–16. 10.1007/s10072-010-0439-6 20953810

[6] Rusina R, Ridzon P, Kulist'ák P, Keller O, Bartos A, Buncová M, et al Relationship between ALS and the degree of cognitive impairment, markers of neurodegeneration and predictors for poor outcome. A prospective study. European Journal of Neurology 2010;17:23–30. 10.1111/j.1468-1331.2009.02717.x 19572947

[7] Phukan J, Elamin M, Bede P, Jordan N, Gallagher L, Byrne S, et al The syndrome of cognitive impairment in amyotrophic lateral sclerosis: a population-based study. Journal of Neurology, Neurosurgery & Psychiatry 2011;83:102–8. 10.1136/jnnp-2011-300188 21836033

[8] Strong MJ, Grace GM, Freedman M, Lomen-Hoerth C, Woolley S, Goldstein LH, et al Consensus criteria for the diagnosis of frontotemporal cognitive and behavioural syndromes in amyotrophic lateral sclerosis. Amyotroph Lateral Scler 2009;10:131–46. 19462523 10.1080/17482960802654364

[9] Murphy JM, Henry RG, Langmore S, Kramer JH, Miller BL, Lomen-Hoerth C. Continuum of frontal lobe impairment in amyotrophic lateral sclerosis. Arch Neurol 2007;64:530–4. 17420314 10.1001/archneur.64.4.530

[10] Grace GM, Orange JB, Rowe A, Findlater K, Freedman M, Strong MJ. Neuropsychological functioning in PLS: a comparison with ALS. The Canadian Journal of Neurological Sciences 2011;38:88–97. 21156436

[11] Terada T, Obi T, Yoshizumi M, Murai T. Frontal lobe-mediated behavioral changes in amyotrophic lateral sclerosis: Are they independent of physical disabilities? Journal of the Neurological Sciences. 2011;309:136–40. 10.1016/j.jns.2011.06.049 21782199

[12] Amyotrophic lateral sclerosis and frontotemporal dementia: A behavioural and cognitive continuum. 2012;13:102–9. Available: http://eutils.ncbi.nlm.nih.gov/entrez/eutils/elink.fcgi?dbfrom=pubmed&id=22214356&retmode=ref&cmd=prlinks. 10.3109/17482968.2011.639376 22214356

[13] Witgert M, Salamone AR, Strutt AM, Jawaid A, Massman PJ, Bradshaw M, et al Frontal-lobe mediated behavioral dysfunction in amyotrophic lateral sclerosis. Eur J Neurol 2010;17:103–10. 10.1111/j.1468-1331.2009.02801.x 19874396

[14] Girardi A, MacPherson SE, Abrahams S. Deficits in emotional and social cognition in amyotrophic lateral sclerosis. Neuropsychology 2011;25:53–65. 10.1037/a0020357 20919762

[15] Weschler IS, Davison C. Amyotrophic lateral sclerosis with mental symptoms: A clinicopathologic study. Archives of Neurology and Psychiatry 1932;:858–80.

[16] Girardi A, MacPherson SE, Abrahams S. Deficits in emotional and social cognition in amyotrophic lateral sclerosis. Neuropsychology 2011;25:53–65. 10.1037/a0020357 20919762

[17] Cavallo M, Adenzato M, MacPherson SE, Karwig G, Enrici I, Abrahams S. Evidence of social understanding impairment in patients with amyotrophic lateral sclerosis. PLoS ONE 2011;6:e25948 10.1371/journal.pone.0025948 21998727 PMC3187828

[18] Gibbons ZC, Snowden JS, Thompson JC, Happe F, Richardson A, Neary D. Inferring thought and action in motor neurone disease. Neuropsychologia 2007;45:1196–207. 17118410 10.1016/j.neuropsychologia.2006.10.008

[19] Kilner JM. More than one pathway to action understanding. Trends in Cognitive Sciences 2011;15:352–7. 10.1016/j.tics.2011.06.005 21775191 PMC3389781

[20] Rizzolatti G, Sinigaglia C. The functional role of the parieto-frontal mirror circuit: interpretations and misinterpretations. Nat Rev Neurosci 2010;11:264–74. 10.1038/nrn2805 20216547

[21] Hesslow G. Conscious thought as simulation of behaviour and perception. Trends in Cognitive Sciences 2002;6:242–7. 12039605 10.1016/s1364-6613(02)01913-7

[22] Rizzolatti G, Fadiga L, Gallese V, Fogassi L. Premotor cortex and the recognition of motor actions. Brain Res Cogn Brain Res 1996;3:131–41. 8713554 10.1016/0926-6410(95)00038-0

[23] Gallese V, Fadiga L, Fogassi L, Rizzolatti G. Action recognition in the premotor cortex. Brain 1996;119 (Pt 2):593–609. 8800951 10.1093/brain/119.2.593

[24] Gallese V, Goldman A. Mirror neurons and the simulation theory of mind-reading. Trends in Cognitive Sciences 1998;2:493–501. 21227300 10.1016/s1364-6613(98)01262-5

[25] di Pellegrino G, Fadiga L, Fogassi L, Gallese V, Rizzolatti G. Understanding motor events: a neurophysiological study. Exp Brain Res 1992;91:176–80. 1301372 10.1007/BF00230027

[26] Cattaneo L, Rizzolatti G. The mirror neuron system. Arch Neurol 2009;66:557–60. 10.1001/archneurol.2009.41 19433654

[27] Molenberghs P, Hayward L, Mattingley JB, Cunnington R. Activation patterns during action observation are modulated by context in mirror system areas. NeuroImage 2012;59:608–15. 10.1016/j.neuroimage.2011.07.080 21840404

[28] Iacoboni M, Molnar-Szakacs I, Gallese V, Buccino G, Mazziotta JC, Rizzolatti G. Grasping the Intentions of Others with One's Own Mirror Neuron System. Plos Biol 2005;3:e79 15736981 10.1371/journal.pbio.0030079 PMC1044835

[29] Fadiga L, Fogassi L, Pavesi G, Rizzolatti G. Motor facilitation during action observation: a magnetic stimulation study. Journal of Neurophysiology 1995;73:2608–11. 7666169 10.1152/jn.1995.73.6.2608

[30] Keysers C, Gazzola V. Social neuroscience: mirror neurons recorded in humans. Curr Biol 2010;20:R353–4. 10.1016/j.cub.2010.03.013 21749952

[31] Eisen A, Turner MR, Lemon R. Tools and talk: an evolutionary perspective on the functional deficits associated with amyotrophic lateral sclerosis. Muscle Nerve 2014;49:469–77. 10.1002/mus.24132 24273101

[32] Jelsone-Swain L, Welsh RC. Mirror neuron function in ALS: A preliminary study. In: Benatar M, editor. Miami: 2012.

[33] van Veluw SJ, Chance SA. Differentiating between self and others: an ALE meta-analysis of fMRI studies of self-recognition and theory of mind. Brain Imaging Behav 2014;8:24–38. 10.1007/s11682-013-9266-8 24535033

[34] Sirigu A, Daprati E, Pradat-Diehl P, Franck N, Jeannerod M. Perception of self-generated movement following left parietal lesion. Brain 1999;122 (Pt 10):1867–74. 10506089 10.1093/brain/122.10.1867

[35] Jeannerod M. Neural simulation of action: a unifying mechanism for motor cognition. NeuroImage 2001;14:S103–9. 11373140 10.1006/nimg.2001.0832

[36] Cattaneo L, Caruana F, Jezzini A, Rizzolatti G. Representation of goal and movements without overt motor behavior in the human motor cortex: a transcranial magnetic stimulation study. J. Neurosci. 2009;29:11134–8. 10.1523/JNEUROSCI.2605-09.2009 19741119 PMC6665924

[37] Rizzolatti G, Fabbri-Destro M, Cattaneo L. Mirror neurons and their clinical relevance. Nat Clin Pract Neurol 2009;5:24–34. 10.1038/ncpneuro0990 19129788

[38] Performance of the Amyotrophic Lateral Sclerosis Functional Rating Scale (ALSFRS) in multicenter clinical trials. 1997;152 Suppl 1:S1–9. Available: http://eutils.ncbi.nlm.nih.gov/entrez/eutils/elink.fcgi?dbfrom=pubmed&id=9419047&retmode=ref&cmd=prlinks. 10.1016/s0022-510x(97)00237-2 9419047

[39] Detecting frontotemporal dysfunction in ALS: utility of the ALS Cognitive Behavioral Screen (ALS-CBS). 2010;11:303–11. Available: http://eutils.ncbi.nlm.nih.gov/entrez/eutils/elink.fcgi?dbfrom=pubmed&id=20433413&retmode=ref&cmd=prlinks. 10.3109/17482961003727954 20433413

[40] Nasreddine ZS, Phillips NA, Bedirian V, Charbonneau S, Whitehead V, Collin I, et al The Montreal Cognitive Assessment, MoCA: a brief screening tool for mild cognitive impairment. J Am Geriatr Soc 2005;53:695–9. 15817019 10.1111/j.1532-5415.2005.53221.x

[41] The verbal fluency index: Dutch normative data for cognitive testing in ALS. 2014;15:388–91. 10.3109/21678421.2014.906620 24862654

[42] Verbal fluency and executive dysfunction in amyotrophic lateral sclerosis (ALS). 2000;38:734–47. Available: http://eutils.ncbi.nlm.nih.gov/entrez/eutils/elink.fcgi?dbfrom=pubmed&id=10689049&retmode=ref&cmd=prlinks. 10.1016/s0028-3932(99)00146-3 10689049

[43] Baron-Cohen S, Wheelwright S, Hill J, Raste Y, Plumb I. The “Reading the Mind in the Eyes” Test revised version: a study with normal adults, and adults with Asperger syndrome or high-functioning autism. J Child Psychol Psychiatry 2001;42:241–51. 11280420

[44] Woolley SC, York MK, Moore DH, Strutt AM, Murphy J, Schulz PE, et al Detecting frontotemporal dysfunction in ALS: utility of the ALS Cognitive Behavioral Screen (ALS-CBS). Amyotroph Lateral Scler 2010;11:303–11. 10.3109/17482961003727954 20433413

[45] Newsom-Davis IC, Abrahams S, Goldstein LH, Leigh PN. The emotional lability questionnaire: a new measure of emotional lability in amyotrophic lateral sclerosis. Journal of the Neurological Sciences 1999;169:22–5. 10540003 10.1016/s0022-510x(99)00211-7

[46] Yesavage JA. Geriatric Depression Scale. Psychopharmacol Bull 1988;24:709–11. 3249773

[47] Rizzolatti G. The mirror neuron system and its function in humans. Anat Embryol (Berl) 2005;210:419–21. 16222545 10.1007/s00429-005-0039-z

[48] Konrad C, Henningsen H, Bremer J, Mock B, Deppe M, Buchinger C, et al Pattern of cortical reorganization in amyotrophic lateral sclerosis: a functional magnetic resonance imaging study. Exp Brain Res 2002;143:51–6. 11907690 10.1007/s00221-001-0981-9

[49] Higuchi S, Holle H, Roberts N, Eickhoff SB, Vogt S. Imitation and observational learning of hand actions: prefrontal involvement and connectivity. NeuroImage 2012;59:1668–83. 10.1016/j.neuroimage.2011.09.021 21983182

[50] Schoenfeld MA, Tempelmann C, Gaul C, Kuhnel GR, Duzel E, Hopf J-M, et al Functional motor compensation in amyotrophic lateral sclerosis. J Neurol 2005;252:944–52. 15750701 10.1007/s00415-005-0787-y

[51] Kollewe K, Munte TF, Samii A, Dengler R, Petri S, Mohammadi B. Patterns of cortical activity differ in ALS patients with limb and/or bulbar involvement depending on motor tasks. J Neurol 2011;258:804–10. 10.1007/s00415-010-5842-7 21128080

[52] Stoppel CM, Vielhaber S, Eckart C, Machts J, Kaufmann J, Heinze H-J, et al Structural and functional hallmarks of amyotrophic lateral sclerosis progression in motor- and memory-related brain regions. Neuroimage Clin 2014;5:277–90. 10.1016/j.nicl.2014.07.007 25161894 PMC4141983

[53] Pineda JA. Sensorimotor cortex as a critical component of an “extended” mirror neuron system: Does it solve the development, correspondence, and control problems in mirroring? Behav Brain Funct 2008;4:47 10.1186/1744-9081-4-47 18928566 PMC2577683

[54] Maldjian JA, Laurienti PJ, Kraft RA, Burdette JH. An automated method for neuroanatomic and cytoarchitectonic atlas-based interrogation of fMRI data sets. NeuroImage 2003;19:1233–9. 12880848 10.1016/s1053-8119(03)00169-1

[55] Gobbini MI, Koralek AC, Bryan RE, Montgomery KJ, Haxby JV. Two takes on the social brain: a comparison of theory of mind tasks. J Cogn Neurosci 2007;19:1803–14. 17958483 10.1162/jocn.2007.19.11.1803

[56] Montgomery KJ, Isenberg N, Haxby JV. Communicative hand gestures and object-directed hand movements activated the mirror neuron system. Soc Cogn Affect Neurosci 2007;2:114–22. 10.1093/scan/nsm004 18985130 PMC2555455

[57] Iacoboni M, Woods RP, Brass M, Bekkering H, Mazziotta JC, Rizzolatti G. Cortical mechanisms of human imitation. Science 1999;286:2526–8. 10617472 10.1126/science.286.5449.2526

[58] Fabbri-Destro M, Rizzolatti G. Mirror Neurons and Mirror Systems in Monkeys and Humans. Physiology 2008;23:171–9. 10.1152/physiol.00004.2008 18556470

[59] Lulé D, Diekmann V, Kassubek J, Kurt A, Birbaumer N, Ludolph AC, et al Cortical plasticity in amyotrophic lateral sclerosis: motor imagery and function. Neurorehabil Neural Repair 2007;21:518–26. 17476000 10.1177/1545968307300698

[60] Poujois A, Schneider FC, Faillenot I, Camdessanche J-P, Vandenberghe N, Thomas-Anterion C, et al Brain plasticity in the motor network is correlated with disease progression in amyotrophic lateral sclerosis. Hum Brain Mapp 2013;34:2391–401. 10.1002/hbm.22070 22461315 PMC6870334

[61] Fiori F, Sedda A, Ferre ER, Toraldo A, Querzola M, Pasotti F, et al Exploring motor and visual imagery in Amyotrophic Lateral Sclerosis. Exp Brain Res 2013;226:537–47. 10.1007/s00221-013-3465-9 23503773

[62] Caspers S, Zilles K, Laird AR, Eickhoff SB. ALE meta-analysis of action observation and imitation in the human brain. NeuroImage 2010;50:1148–67. 10.1016/j.neuroimage.2009.12.112 20056149 PMC4981639

[63] Vogt S, Rienzo FD, Collet C, Collins A, Guillot A. Multiple roles of motor imagery during action observation. Front Hum Neurosci 2013;7:807 10.3389/fnhum.2013.00807 24324428 PMC3839009

[64] Kelly AMC, Garavan H. Human functional neuroimaging of brain changes associated with practice. Cerebral Cortex 2005;15:1089–102. 15616134 10.1093/cercor/bhi005

[65] Babiloni C, Del Percio C, Rossini PM, Marzano N, Iacoboni M, Infarinato F, et al Judgment of actions in experts: a high-resolution EEG study in elite athletes. NeuroImage 2009;45:512–21. 10.1016/j.neuroimage.2008.11.035 19111623

[66] Kew JJ, Leigh PN, Playford ED, Passingham RE, Goldstein LH, Frackowiak RS, et al Cortical function in amyotrophic lateral sclerosis. A positron emission tomography study. Brain 1993;116 (Pt 3):655–80. 8513396 10.1093/brain/116.3.655

[67] Mohammadi B, Kollewe K, Samii A, Dengler R, Munte TF. Functional neuroimaging at different disease stages reveals distinct phases of neuroplastic changes in amyotrophic lateral sclerosis. Hum Brain Mapp 2011;32:750–8. 10.1002/hbm.21064 20836159 PMC6870014

[68] Tessitore A, Esposito F, Monsurro MR, Graziano S, Panza D, Russo A, et al Subcortical motor plasticity in patients with sporadic ALS: An fMRI study. Brain Res Bull 2006;69:489–94. 16647577 10.1016/j.brainresbull.2006.01.013

[69] Filimon F, Nelson JD, Hagler DJ, Sereno MI. Human cortical representations for reaching: mirror neurons for execution, observation, and imagery. NeuroImage 2007;37:1315–28. 17689268 10.1016/j.neuroimage.2007.06.008 PMC2045689

[70] Meiri H, Sela I, Nesher P, Izzetoglu M, Izzetoglu K, Onaral B, et al Frontal lobe role in simple arithmetic calculations: An fNIR study. Neuroscience Letters 2012;510:43–7. 10.1016/j.neulet.2011.12.066 22260794

[71] The Relationship between Depressive Symptoms, Disease State, and Cognition in Amyotrophic Lateral Sclerosis. 2012;3:542. Available: http://eutils.ncbi.nlm.nih.gov/entrez/eutils/elink.fcgi?dbfrom=pubmed&id=23411492&retmode=ref&cmd=prlinks. 10.3389/fpsyg.2012.00542 PMC3571885 23411492

[72] Palmieri A, Mento G, Calvo V, Querin G, D'Ascenzo C, Volpato C, et al. Female gender doubles executive dysfunction risk in ALS: a case-control study in 165 patients. Journal of Neurology, Neurosurgery & Psychiatry 2014; 10.1136/jnnp-2014-307654 25063584

[73] Raaphorst J, Beeldman E, Schmand B, Berkhout J, Linssen WHJP, van den Berg LH, et al The ALS-FTD-Q: a new screening tool for behavioral disturbances in ALS. Neurology 2012;79:1377–83. 22972650 10.1212/WNL.0b013e31826c1aa1

[74] Lulé D, Diekmann V, Anders S, Kassubek J, Kubler A, Ludolph AC, et al Brain responses to emotional stimuli in patients with amyotrophic lateral sclerosis (ALS). J Neurol 2007;254:519–27. 17401515 10.1007/s00415-006-0409-3

[75] Schmolck H, Mosnik D, Schulz P. Rating the approachability of faces in ALS. Neurology 2007;69:2232–5. 18071142 10.1212/01.wnl.0000296001.16603.b3

[76] Zimmerman EK, Eslinger PJ, Simmons Z, Barrett AM. Emotional perception deficits in amyotrophic lateral sclerosis. Cogn Behav Neurol 2007;20:79–82. 17558250 10.1097/WNN.0b013e31804c700b PMC1905862

[77] Lulé D, Diekmann V, Muller H-P, Kassubek J, Ludolph AC, Birbaumer N. Neuroimaging of multimodal sensory stimulation in amyotrophic lateral sclerosis. Journal of Neurology, Neurosurgery & Psychiatry 2010;81:899–906. 10.1136/jnnp.2009.192260 20543183

[78] Bak TH, Chandran S. What wires together dies together: Verbs, actions and neurodegeneration in motor neuron disease. CORTEX 2012;48:936–44. 10.1016/j.cortex.2011.07.008 21924711

[79] York C, Olm C, Boller A, McCluskey L, Elman L, Haley J, et al Action verb comprehension in amyotrophic lateral sclerosis and Parkinson's disease. J Neurol 2014;261:1073–9. 10.1007/s00415-014-7314-y 24676939 PMC4074280

[80] Hillis AE, Oh S, Ken L. Deterioration of naming nouns versus verbs in primary progressive aphasia. Ann Neurol 2004;55:268–75. 14755731 10.1002/ana.10812

[81] Grossman M, Anderson C, Khan A, Avants B, Elman L, McCluskey L. Impaired action knowledge in amyotrophic lateral sclerosis. Neurology 2008;71:1396–401. 10.1212/01.wnl.0000319701.50168.8c 18784377 PMC2676962

[82] Buccino G. Action observation treatment: a novel tool in neurorehabilitation. Philos Trans R Soc Lond B Biol Sci 2014;369:20130185 10.1098/rstb.2013.0185 24778380 PMC4006186

